# Total-body low-dose irradiation of mice induces neither learning disability and memory impairment in Morris water maze test nor Alzheimer's disease-like pathogensis in the brain

**DOI:** 10.1093/jrr/rrt189

**Published:** 2014-03

**Authors:** Bing Wang, Kaoru Tanaka, Bin Ji, Maiko Ono, Yaqun Fang, Yasuharu Ninomiya, Kouichi Maruyama, Nakako Izumi-Nakajima, Nasrin Begum, Makoto Higuchi, Akira Fujimori, Yoshihiko Uehara, Tetsuo Nakajima, Tetsuya Suhara, Tetsuya Ono, Mitsuru Nenoi

**Affiliations:** 1 National Institute of Radiological Sciences, Anagawa 4-9-1, Inage-ku, Chiba 263-8555, Japan; 2 Centre for Nuclear Medicine and Ultrasound, Rajshahi 6000, Bangladesh; 3 Tohoku University, Sendai 980-8575, Japan; 4 Institute for Environmental Sciences, Aomori 039-3212, Japan

**Keywords:** total-body irradiation, low dose, Alzheimer's disease, Morris water maze test, Alzheimer's disease-like pathogenesis, mice

## Abstract

**Purpose:** Alzheimer's disease (AD) is the most common form of dementia, while its cause and progression are not well understood [ 1]. The possible cognitive and behavioral consequences induced by low-dose radiation are of great concern as humans are exposed to ionizing radiations from various sources including medical diagnosis [ 2]. A recent study in mice reported early transcriptional response in brain to low-dose X-rays (0.10 Gy) suggesting alterations of molecular networks and pathways associated with cognitive functions, advanced aging and AD. The present study is to investigate the late pathological, cognitive and behavioral consequences induced by low-dose radiation.

**Materials and methods:** C57BL/6J mice were total-body irradiated with an acute dose from X-rays (0.10 Gy) or carbon ions (0.05 or 0.10 Gy). The hippocampus was collected and the expression of 84 AD-related genes was analyzed. Morris water maze test was applied to the measurement of the learning ability and memory of the animals. Amyloid imaging with positron emission tomography were performed to detect the accumulation of fibrillary amyloid β peptide (Aβ), and characteristic pathologies of AD were examined with immunohistochemical staining of amyloid precursor protein (APP), Aβ, tau, and phosphorylated tau.

**Results:** For the transcriptional studies, results showed that a few genes out of 84 AD-related genes were significantly up-regulated at 4 h after irradiation and the other genes had no marked change; on the other hand, a few other genes showed a significant down-regulation, while the other genes had no marked change at 1 year after irradiation. For the behavioral studies, no significant difference on learning ability and memory was observed at 1 and 2 years after irradiation. Imaging and immunohistochemical staining showed no change in the accumulation of fibrillar amyloid and the expression of APP, Aβ, tau and phosphorylated tau were detectable in the animals 4 months and 2 years after irradiation.

**Conclusion:** These findings suggest that total-body irradiation at a dose of 0.10 Gy could hardly induce significant early or late transcriptional alterations in most of the AD-related genes in the hippocampus, learning disability and memory impairment, and AD-like pathological change in the brain in mice [ 3].
